# Bone Augmentation for Implant Placement: Recent Advances

**DOI:** 10.1155/2022/8900940

**Published:** 2022-03-27

**Authors:** Van Viet Dam, Hai Anh Trinh, Dinesh Rokaya, Dinh Hai Trinh

**Affiliations:** ^1^Department of Implantology, Ha Noi National Hospital of Odonto-stomatology, Hanoi, Vietnam; ^2^VNU School of Medicine and Pharmacy, Vietnam National University, Hanoi, Vietnam; ^3^Walailak University International College of Dentistry, Walailak University, Bangkok 10400, Thailand

## Abstract

There are various advancements in biomaterials and methods for bone augmentation. This article aims to review the recent advances in bone augmentation for dental implants. Relevant articles on bone augmentation for dental implants were searched in PubMed/Medline, Scopus, Google Scholar, and Science Direct published in English literature published between January 1996 and March 2021. Relevant studies on bone grafts for dental implants were included and critically analyzed in this review. Various biomaterials can be used to augment bone for implant placement. Each graft procedure has advantages and disadvantages in each clinical application and needs to choose the graft material with a high success rate and less morbidity.

## 1. Introduction

Dental aesthetics is one of the prime objectives of prosthodontic treatment. Facial aesthetics as macroaesthetics and that of peri-oral and dental tissues understood, respectively, as micro- and miniaesthetics play a fundamental role in the preliminary aesthetic and functional evaluation of the patient [1–4]. The dental implant has played an important role in oral rehabilitation, restorative dentistry, and maxilla-facial reconstruction. Currently, the use of dental implants has increased due to their high success and survival rates [5–7]. According to Straumann, approximately 10.7 million implants are placed annually all over the world [8]. Dental implants are used in the replacement of missing teeth or provide retention and support for prostheses [9, 10]. In particular, prosthetically guided approaches to implant insertion, fully digital, are currently a possible and recommended alternative [11, 12]. Composition and roughness play an important role in implant-tissue interaction and osseointegration [13–15]. But due to insufficient bone or bone defects in the maxilla and mandible, it is often difficult for implant placement. In such situations, bone grafts play a vital role in the restoration of bone.

**Figure 1:**
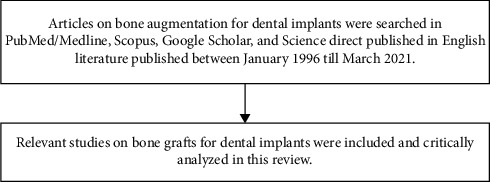
Schematic diagram of the method for the selection of articles.

Bone regeneration is rapidly evolving to treat various defects in the human body. There are various advancements in biomaterials and methods for bone augmentation. The outcome of the biodegradable scaffold is dependent on the interdisciplinary collaboration among clinicians, bioengineers, and materials scientists [16]. The use of different scaffold material types, stem cells, and growth factors shows promise in regenerative treatment interventions for maxillofacial defects [16–19]. This article aims to review the recent advances in bone augmentation for dental implants. Articles on bone augmentation for dental implants were searched in PubMed/Medline, Scopus, Google Scholar, and Science Direct published in English literature published between January 1996 and March 2021 (Figure 1). Relevant studies on bone grafts for dental implants were included and critically analyzed in this review.

## 2. Bone Grafts and Their Types

Biomaterials are natural or synthetic substances and help to repair, augment, or replace any tissue or organ of the body [20, 21]. Bone grafts are the type of biomaterials that are used to substitute bone defects and recover atrophic bone regions [22]. They are major components in maxillofacial surgery and implantology. A bone graft is also needed for 1 of every 4 implants [23]. They are generally used as scaffolds and fillers to accelerate bone augmentation as they act as a reservoir for new bone formation. They are bioresorbable with no antigen-antibody reaction [24, 25]. It is important to have a clear and detailed understanding of the fundamentals in regenerative science for successful outcomes in bone grafting [26].

Different bone grafts are used in clinical practice, and the classifications are based on composition, physical properties, and other parameters [4]. Classification of bone grafts based on composition is shown in Table 1 [27] which are based on allograft, factor, cell, ceramic, and polymer. Above gone grafts can be used alone or in combination with other grafts. The chronological classification divides the bone substitutes into 5 divisions: xenograft, allograft, and autogenic bone; allogenic bone; natural bone matrix containing growth factors; tissue-engineered; and gene-activated bone grafts [28]. Autogenous bone is regarded as the gold standard because of its biocompatibility, osteoconductive, osteoinductive, and osteogenic properties [28–33].

## 3. Fibula Free Flap and Iliac Crest Flap

Segmental or partial mandibular defects from trauma or resective surgery result in various degrees of skin, mucosa, or soft tissue loss [34]. The use of free bone flaps such as the fibula, iliac crest, and scapula has revolutionized the maxillofacial rehabilitation in extensive mandibular defects [35]. Well-vascularized bone with soft tissues is used in repairing and reconstructing maxilla-mandibular defects achieving morphological and functional goals [36]. Ideally, the ideal flap should be vascularized bone with adequate height and length that can be shaped to match the original mandible.

**Figure 2:**
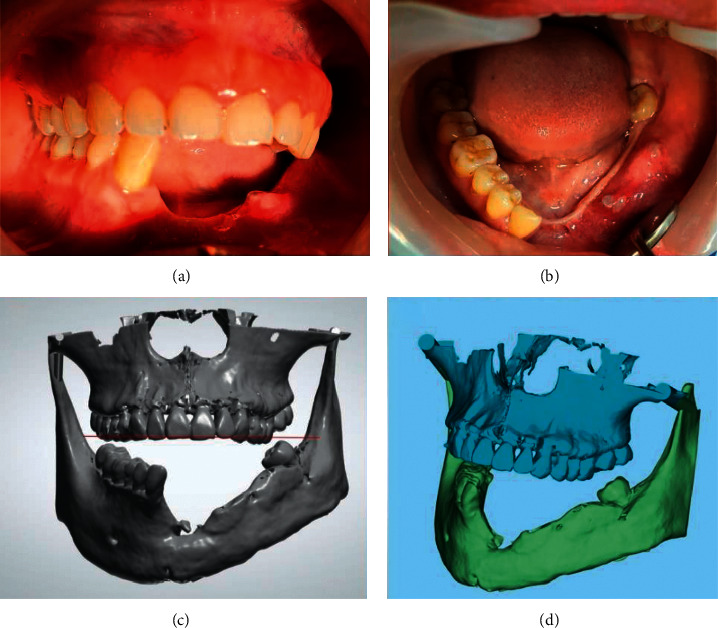
Fibula free flap in the mandibular arch: clinical pictures (a, b) and 3D views (c, d).

Figure 2 shows the rehabilitation of mandibular defects with a fibula graft in a female patient following the resection of ameloblastoma. A fibula free flap (FFF) was done to restore the bone defect and receive the prostheses. The advantages of FFF for the reconstruction of the mandible include an adequate length of bone, the possibility of using a skin paddle, and donor site low morbidity [35]. However, the disadvantage is that it is difficult to reconstruct large soft tissue defects and reduction of bone vascularization following many osteotomies.

In a clinical study by Yilmaz et al. [34], they performed 37 mandibular reconstructions involving skin and/or mucosa: 16 out of 24 patients with iliac crest flap and 3 out of 13 patients with FFF. They found that FFF showed better oral continence, aesthetic facial appearance, and social activities with less complication rate compared to the iliac crest flap.

Lonie et al. [37] did a systematic review of iliac free flaps versus FFFs for mandibular defect reconstruction. There was a significant delay in healing and breakdown of the suture line in the iliac flap group but higher donor site complications in FFF. Osseointegrated dental implant loss in FFF (5.3%) was higher than in iliac flaps (1.7%). The flap loss in FFF and the iliac free flap showed no significant difference. Although they concluded that both iliac free flaps and FFF can be considered in the reconstruction of the mandible, the iliac crest can be considered as the 1^st^ choice for the reconstruction of the body or angle defects in the mandible or defects needing greater thickness of soft tissue, whereas the FFF can be considered as the 1^st^ choice when the bony length is essential as in the case of total or subtotal mandibulectomy.

In addition, a mandibular reconstruction should support the dental implants for total prosthetic rehabilitation [34, 38]. Vascularized fibula grafts are appropriate primary and secondary implantation for prosthetic restoration of the mandibular defects [39, 40]. Wei et al. [40] mentioned that inserting dental implants after some months following mandibular reconstruction using vascularized bone grafts is successful. Hypothetically, primary placement of implants in the mandible presents higher success in a shorter period in oral rehabilitation. Still, the success of a dental implant depends on the condition of the mandible and the history of radiotherapy. In addition, soft tissue and bone needs, the use of implants, and several surgeries are important for the planning.

The FFF contains thick cortical bone with a fatty marrow, but the marrow limits bone stock. Recently, bone-impacted FFF (BIFFF), a novel technique, has been used in which the autologous bone is compacted into the FFF marrow space which increases the density of implant site for the dental implant and this increases the long-term success rate of a dental implant with no or less risk of complications [41, 42]. In addition, bone marrow can be centrifuged to generate mesenchymal stem cell concentrates for better osseointegration. Furthermore, in vitro culture can produce progenitor cells [43]. In maxillofacial rehabilitation, both the iliac crest and FFF are commonly used to harvest bone for a dental implant or the reconstruction of jaw defects [22].

**Figure 3:**
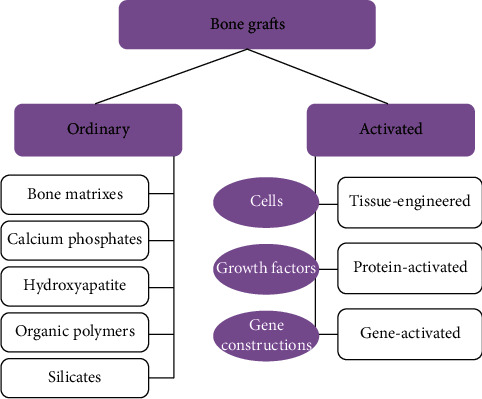
Types of ordinary or activated bone grafts [22].

The bone grafts can be ordinary or activated as shown in Figure 3 based on their composition and biological effects. The osteoinductivity and osteogenicity of activated osteoplastic biomaterials allow the replacement of large bone defects.

## 4. Combination of Bone Grafts

The combination of autogenous bone graft with deproteinized bovine bone has shown better results because of its osteogenic property [44–48]. Kim et al. [24] assessed the bone formation and stability of grafts with autogenous bone and Bio-Oss at different amounts in rabbit calvaria. They concluded that the Bio-Oss either alone or with the 25% autogenous bone showed better stability compared to autogenous bone alone. Similarly, another study [29] compared the histology of bone filled with Bio-Oss, PerioGlas, or Ostim-Paste in the rabbit tibiae defect. They found that the implants placed in all 3 grafting materials presented better osseointegration due to osteoconductive because of the formation of the mineralized bone bridge extending from the cortical plate to the surface of the implant compared to the nongrafted bone.

Thuaksuban et al. [47] compared the clinical outcomes of composite autogenous bone + deproteinized bone from bovine and autogenous bone alone to repair a cleft palate. Group I consisted of autogenous cancellous bone grafts harvested from the anterior iliac crests. Group II consisted of a composite of deproteinized bovine bone and autogenous cancellous bone (1 : 1 proportion by volume). The operation time, blood loss, postoperative pain, hospital stay, and recovery time were more in Group I than in Group II.

Various materials and techniques are being used to create the structural base of osseous tissue to support dental implants. Aghaloo and Moy [33] did a systematic review to identify the successful technique to provide the alveolar bone for the success of the dental implant. They mentioned that the bone augmentation in the alveolar ridge is technique-sensitive and does not have detailed documentation or long-term follow-up studies, except for GBR.

## 5. Bone Grafts Containing Growth Factors

At present, bone grafts with scaffold and growth factors can provide a successful osteoinductive effect. Such various products for clinical use are shown in Table 2 [22].

**Figure 4:**
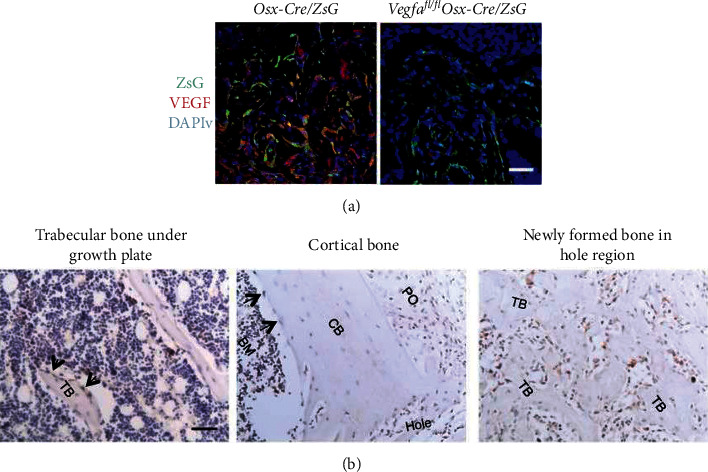
Osteoblastic lineage cells at the bone-repair site as a source of VEGF at postsurgery day 7 in WT mice. (a) A low density of anti-VEGF staining (red) in Vegfa^fl/fl^ Osx-Cre/ZsG mice (6.1%) compared with Osx-Cre/ZsG mice (15.5%). (b) VEGF in cortical bone, trabecular bone, and the newly formed bone within cortical defects. Black arrows show the VEGF-expressing osteoblast lining. TB = trabecular bone and CB = endosteum of cortical bone [52].

Various bone substitutes with growth factors are being developed such as recombinant BMP-2 [49, 50] or VEGF [51, 52]. The osteoblastic lineage cells are an important source of VEGF at the bone-repair site (Figure 4).

The combination of bone substitutes with growth factors with several factors such as angiogenic and osteogenic in the scaffold, for example, VEGF and BMP-2, [53] causes a prolonged release of therapeutic proteins from the matrix with biodegradation [54, 55] or the growth factors' encapsulation into polymer microspheres [56]. The structure of growth factors can be changed using site-directed mutagenesis creating “mutant” molecules causing osteogenesis. Kasten et al. modified the differentiation factor-5 (GDF-5) by binding BMP-2 with specific receptors in its sequence and the GDF-5 molecule showed the properties typical of BMP-2 [57, 58]. Their action is long term than the bone substitutes with growth factors due to the expression of gene constructions being delivered to target cells.

## 6. Gene-Activated Bone Grafts

The active agent of gene-activated grafts is nucleic acids, and they are directly related to gene therapy, such as gene-therapeutic drugs [22]. In addition, gene-activated bone substitute is combined using chemical binding, adjuvants, or fusion of nucleic acids into the graft scaffold [59]. The efficacy of these products is determined by the osteoinduction by gene construction and osteoconduction by scaffold.

There are 2 stages in the osteoinductive action of gene-activated bone grafts: specific and nonspecific. The specific action contains protein regulatory molecules produced by transfected cells which act as bioreactors of therapeutic proteins. The nonspecific action is associated with the release of nucleic acids from the graft scaffold, delivery to the cells, and expression. The gene-activated bone graft presents significantly higher efficacy compared to the substitutes containing growth factors [60].

## 7. Limitations and Future Perspectives

Bone grafts have certain shortcomings, especially which have growth factors. Firstly, protein molecules in the body undergo rapid biodegradation from exudation and proteolytic enzymes limiting their osteoinductive action [22]. Second, the therapeutic protein acts short term, and it is difficult for controlled release. In addition, newer technologies such as growth factors using recombinant growth factors also have limitations in using such biomaterials in surgery for early release in wound healing. Another emerging technique for the delivery of growth factors is gene therapy [61], where genetic material is transferred into the genome to produce specific action through a functional protein, such as BMP. The biodegradable scaffolds are developed to maintain space to promote vascular ingrowth, and cell adhesion [43]. Various techniques can be used to study the bone structures, such as cell staining, infrared absorption spectroscopy, and CBCT [52, 62–65]. In addition, it is important to consider technological evolution to reduce the damage and side effects of necessary diagnostic tests. This can be done by specifying the difference between radiation-free and nonradiation in evaluating the effects [66]. The use of ultrasound in dentistry can represent a radiation-free alternative to the other most used exams.

Similarly, digital technologies are at the forefront for integrating 3D and 4D printing with other technologies that can be applied in implant dentistry. A CBCT of the jaw can produce virtual planning of the reconstruction using software and can produce a 3D model of the jaw for reconstruction [62, 63]. Furthermore, allogeneic graft and xenograft block bone grafts may be milled to make custom-fit [67]. In addition, custom-made resorbable scaffolds or custom titanium meshes can be fabricated containing growth factor-enhanced grafts routinely using a 3D printer [43, 68].

## 8. Conclusion

There are various advancements in biomaterials and methods for bone augmentation. Various biomaterials can be used to augment bone for implant placement. No single biomaterial or clinical technique is ideal, and the clinicians need to decide the suitable approach which can provide suitable results with less complication. Each graft procedure has advantages and disadvantages and should use the material with a high success rate and less morbidity. The use of different scaffold material types, stem cells, and growth factors show promise in regenerative treatment interventions for maxillofacial defects.

## Figures and Tables

**Table 1:** Classification of bone grafts based on the composition.

SN	Types of bone graft	Description and examples
1	Allograft based	Allograft bone, such as grafton and orthoblast
2	Factor based	Natural and recombinant growth factors, such as transforming growth factor-beta (TGF-beta), platelet-derived growth factor (PDGF), bone morphogenic protein (BMP), and fibroblast growth factors (FGF)
3	Cell based	Cells generate new tissue, such as mesenchymal stem cells
4	Ceramic based	Calcium phosphate, calcium sulphate, and bioglass, such as Osteograf, Osteoset, and Proosteon
5	Polymer based	Biodegradable and nondegradable polymers, such as open porosity polylactic acid polymer

**Table 2:** Various growth factors used with bone grafts.

Growth factor	Main constituent	Producer
Emdogain	Enamel matrix proteins	Straumann, Germany
OP-1	Recombinant BMP-7	Stryker Biotech, USA
Infuse	Recombinant BMP-2	Medtronic, USA
GEM21S	Bone graft with recombinant PDGF-ВВ	BioMimetic Therapeutics Inc., USA
i-Factor putty	Protein P-15 (ligand for integrins *α*2*β*1)	Cerapedalloics, USA

## Data Availability

The data used to support the findings of this study are available from the corresponding author upon request.
